# Multibody System-Based Adaptive Formation Scheme for Multiple Under-Actuated AUVs

**DOI:** 10.3390/s20071943

**Published:** 2020-03-30

**Authors:** Hai Huang, Qirong Tang, Guocheng Zhang, Tiedong Zhang, Lei Wan, Yongjie Pang

**Affiliations:** 1 National Defense Key Laboratory of Underwater Vehicles Technology, Harbin Engineering University, Harbin 150001, China; haihus@163.com (H.H.); zhangguocheng168@sina.com (G.Z.); wanlei@hrbeu.edu.cn (L.W.); pangyongjie@hrbeu.edu.cn (Y.P.); 2 Laboratory of Robotics and Multibody System, Tongji University, Shanghai 201804, China; qirong.tang@outlook.com

**Keywords:** underwater vehicle formation, limited communication, adaptive formation, multibody system

## Abstract

Underwater vehicles’ coordination and formation have attracted increasing attention since they have great potential for real-world applications. However, such vehicles are usually under-actuated and with very limited communication capabilities. On the basis of the multibody system concept, a multiple autonomous underwater vehicle formation and communication link framework has been established with an adaptive and radial basis function (RBF) strategy. For acoustic communication, a packets transmission scheme with topology and protocol has been investigated on the basis of an acoustic communication framework and transmission model. Moreover, the cooperative localization errors caused by packet loss are estimated through reinforcement learning radial basis function neural networks. Furthermore, in order to realize formation cruising, an adaptive RBF formation scheme with magnitude reduced multi-layered potential energy functions has been designed on the basis of a time-delayed network framework. Finally, simulations and experiments have been extensively performed to validate the effectiveness of the proposed methods.

## 1. Introduction

Currently, Autonomous Underwater Vehicles (AUVs) are increasingly attractive for various underwater tasks such as environmental exploration [1], seabed survey [2], harbor protection [3], and submarine search and rescue [4]. Recent advances in Autonomous Underwater Vehicle (AUV) research have enabled the utilization of multiple AUVs (MAUVs) to realize complex marine missions [5,6,7]. Moreover, although acoustic communications allow the improvement of localization precision, they are still limited with propagating delays and communication channel noise [8]. The upper bound range rate of underwater acoustic channel is 40 km·kb/s [9]. In order to achieve a global objective, each AUV can be taken as a relay and mobile node [10]. Information flow with interconnection topology graphs is necessary for reliable communication [11]. These graphs can have undirected or directed edges for the model of position constraints, information flow, or leader following inter-agent control specifications [12]. Therefore, the MAUVs’ acoustic communication structure should include both communication topology structure [13] and some collision-free data link schemes [14] in order to reduce information congestion and improve flow efficiency [15]. Meng, Shi, and Wang [16] presented a multi-channel scheme based on the multiple access with collision avoidance protocol to improve the network efficiency using code division multiple access (CDMA). Two channels are used for RTS (request to send)/CTS (clear to send) data packets. All nodes in the network are assigned to the same common channel for any packet arrival. One of the disadvantages of this protocol is the centralized nature of the CDMA scheme. In modular and scalable communication architecture, the data infrastructure includes navigation data, tracking data, target data, data request telegrams, etc. [17]. It can be reused, expanded, and bridged for wide area networks. However, the data for transmission is so great that wireless communication was used to assist acoustic communication during the sea trials of two AUVs’ cooperative missions [18]. Guo, Frater, and Ryan [19] proposed an adaptive propagation-delay tolerant media access control (MAC) [20] protocol for an underwater acoustic communication network. The protocol performs an improved handshaking with destination using RTS and CTS frames before transmitting the actual data frame to improve the communication efficiency. In order to further consider the channel blocking effect for simultaneous localization, artificial intelligence-based learning strategies have been employed for controllers [21,22].

In order to realize MAUVs’ formation, the vehicles’ trajectories not only depend on their own dead reckoning and control [23,24], but also depend on group objectives and environmental obstacles. Pramod Abichandani et al. [25] proposed a mixed integer nonlinear programming method for MAUVs’ motion planning under constraint communication. The collision-free trajectories accommodate stricter safety requirements despite intersecting or overlapping paths. Yukun Lin et al. [26] utilized a leader/follower multi-AUV control system to enable the AUVs to drive toward the target through a collision-free path. Mingzhi Chen et al. [27] proposed a novel cooperative hunting algorithm for inhomogeneous MAUVs to achieve quick and active path pursuit and planning. Marcello Farina et al. [28] proposed a distributed predictive control approach for robot coordination. The cost function was defined for rapid exploring formation control [12], coverage sensing, and collision avoidance. However, MAUVs’ formation in unknown environments and navigation in hostile environments [29] are often baffled with vehicle nonlinearity, constraint communications, environmental disturbance, and obstacles. Xiang Cao et al. [30] proposed a target following a cooperative search approach by combining the Glasius bio-inspired neural network with a bio-inspired cascaded tracking control approach to improve their search efficiency and reduce tracking errors. Hao Wang et al. [31] designed a smoothly switching function-based neural network adaptive technique to compensate system uncertainties for cooperative path following. Chengzhi Yuan et al. [32] proposed a learning-based formation scheme for multiple AUVs on heterogeneous nonlinear uncertain dynamics under the virtual leader-following framework, which includes an adaptive observer and a deterministic learning controller. The learned knowledge can be effectively stored in a time-invariant fashion by using radial basis function (RBF) neural networks. However, the formation error and stability not only lie in the controller quality, but they also are affected by the cooperative localization errors caused by packet transmission losses.

This paper proposed an adaptive formation scheme on the basis of multibody system concept, the contributions can be summarized in the following:

1. On the basis of the multibody system concept, the MAUVs’ formation and communication link framework has been established. The connection between AUVs can be viewed as a springs and damping system. An adaptive control strategy has been set up for multiple under-actuated AUVs formation with a desired formation region and magnitude reduced artificial potential function.
2. On the basis of the MAUVs’ formation and communication link framework, the packets transmission scheme has been designed with learning-based multi-layered network topology; the cooperative localization errors caused by packet loss are estimated and modified through reinforcement learning RBF neural networks.
3. On the basis of the MAUVs’ formation and communication link framework, an adaptive RBF formation scheme with magnitude reduced multi-layered potential energy functions has been designed on the basis of the time-delayed network framework. Simulations and experiments have verified the performance of the purposed schemes.

The rest of this study is organized as follows. In Section 2, the MAUVs’ formation and communication link framework will be proposed. An adaptive formation control approach of multiple AUVs will be proposed in Section 3. Simulations and experiments will be discussed and analyzed in Section 4. We will present the conclusion in Section 5.

## 2. MAUVs’ Formation and Communication Link Framework

### 2.1. Multibody-Based Formation Framework

**Figure 1:**
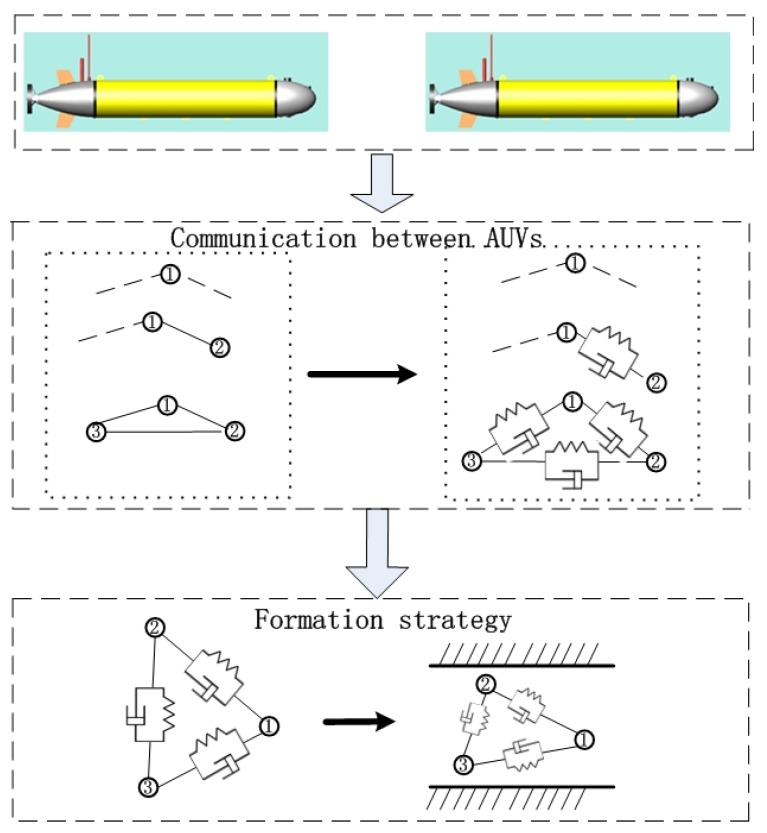
Multibody system based multiple Autonomous Underwater Vehicle (AUV) formation framework.

When MAUVs cruise in the oceanic environment, they can be taken as mobile nodes. In comparison with the nodes of a normal multibody system, the distances between the nodes and their shapes are changing over time. Their connections are soft and established through communications and formation control approaches. However, the multibody system concept can strengthen the bonds and formation robustness between AUVs. From the multibody system concept (see Figure 1), the constraints between adjacent AUVs are designed through a virtual spring and damping system. During the formation process, the distance constraints between the i-th node and the j-th or the k-th node could be modified or from the variation of the external environment during the formation process. Although the formation is expected to maintain the shape, it is still necessary for shape modification for varied external environments, such as obstacles, narrow passages, dangerous zones, and so forth. Therefore, the shape modification could also lead to changes in the node positions and constraints of the multibody system.

### 2.2. Adaptive Communication Protocol

**Figure 2:**
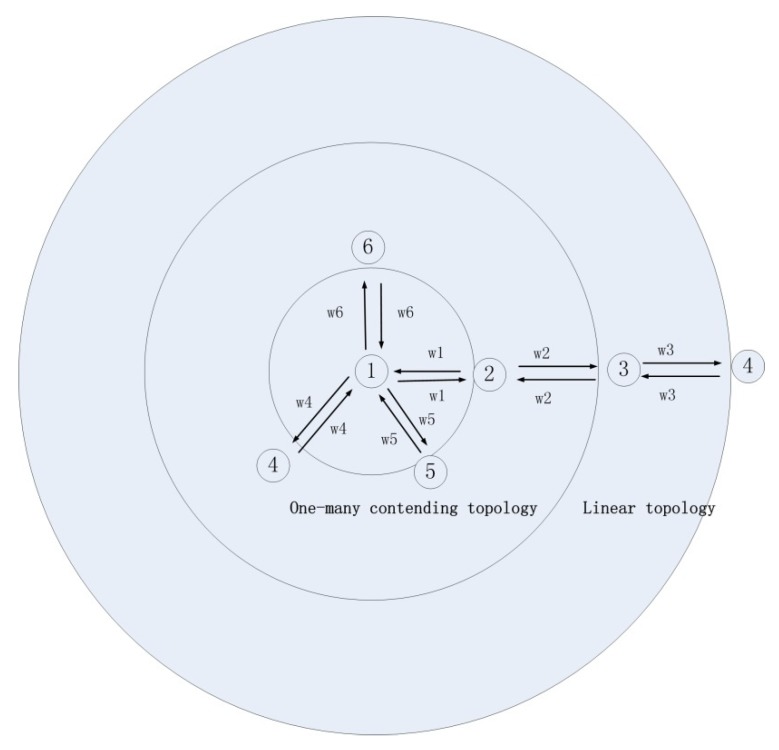
Multiple AUVs (MAUV) network topology.

If we take multiple AUVs’ formation as a multibody system, the mobile AUV nodes should be connected and coordinated over network communication. However, constantly varying the nodes’ distance and transmission latency could lead to the difficulties in data transmission and relative distance observation. Moreover, the energy consumption is correlated with the data transmission distance from source nodes. We adopt a multi-layer-based AUV formation topology according to their relative distance. Linear topology communication is applied for objective AUV nodes far away from the source AUV node while others are closer in the same direction; a one–many contending topology communication is applied for the other leader–follower conditions. The Multiple AUVs network topology is shown in Figure 2.

#### 2.2.1. Protocol for Linear Topology

**Figure 3:**
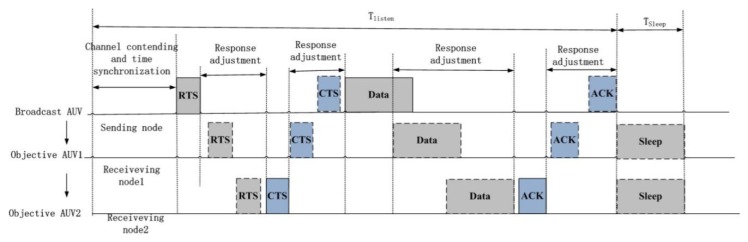
Communication protocol for linear topology.

For communication between source node and objective nodes, the protocol at the data link layer includes four-way handshaking access methods for packets waiting and transmission control such as “RTS”, “CTS”, “Data”, “Acknowledgment for Receiving” (ACK), and “Blocked to Send (BTS)”. The communication process is illustrated in Figure 3.

After channel contention and selection, the source AUV node and objective AUV nodes realize time consensus through broadcasting and answering. The source AUV node will send information to the objective AUV node 2 through objective AUV node 1.

Secondly, the source AUV node will send information to the source node. The format of the data package is {RTS/overtime, node_pos, node_speed, destination_node}, which denotes the data send request, present position, speed, and destined AUV node (in Figure 3, the objective AUV node 2 is supposed as the destined AUV node). When the “RTS” message has been received by the objective AUV node 1, it will be sent to the objective AUV node 2 immediately. At the same time, objective AUV node 1 will be waiting for the “CTS” message from the objective AUV node 2 or a return timeout frame. When the “RTS” message has been received by the objective AUV node 2, it is informed about the forthcoming message, comes into “response adjustment” status, and sends the “CTS” message to the source AUV node through the objective AUV node 1. When the “CTS” message is received by the objective AUV node 1, it will be transmitted to the source AUV node with the format of the data package as {CTS1/overtime, node1_pos, node1_speed, CTS2/overtime, node2_pos, node2_speed}, which denotes the speed and position of objective nodes. When timeout happens, the source AUV node will send the request again or reselect another objective AUV node.

Thirdly, when the “CTS” message is received, “data” will be sent from the source AUV node to the objective AUV node 2 through the objective AUV node 1. When the objective AUV node 1 received “data”, it will come into “Response adjustment” status and send the data package to the objective AUV node 2. After the data has been received, the objective AUV node 2 will return “ACK” to the source node through the objective AUV node 1. The format of the data package is {ACK1/overtime, node1_pos, node1_speed, ACK2/overtime, node2_pos, node2_speed}, which denotes the speed and position of objective nodes. After “ACK” has been received by the source node, the transmission process will terminated.

#### 2.2.2. Protocol for One-Many Contending Topology

**Figure 4:**
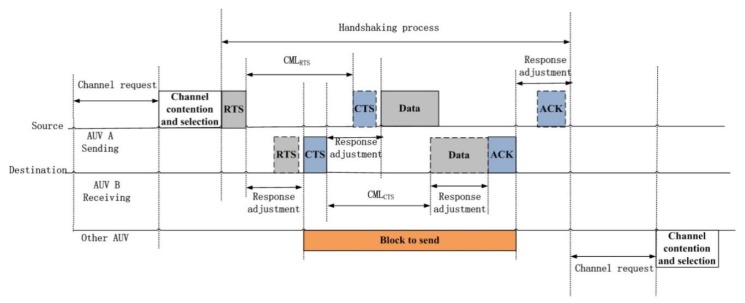
Communication protocol for one–many contending topology.

The protocol includes a four-way handshaking access method for “RTS”, “CTS”, “Data”, and “Acknowledgment for receiving”, as well as “Blocked to Send” packets for waiting control. The “Response adjustment” time includes the time of propagation and process delay. Once a source decides to start transmission through one channel, the handshaking process will start and transmit a “Blocked to Send” to other sources (other AUVs) at the same time (see Figure 4). 

At the first stage, when the RTS frame is received, the destination is notified for the forthcoming transmission. The destination goes to the “Response adjustment” state to receive the packets from its neighbor through the selected channel. A block to send is transmitted to other neighbors so as to alert potential interferers that this channel will be busy for the whole carrying time before it can cause a collision. 

At the second stage, the source waits until receiving either “CTS” or a timeout frame. When a timeout occurs, the source is back to the channel contention and selection state. Obviously, the propagation delay between a frame and its “Response adjustment” is at least equal to the length of the frame to be transmitted/received in it so that the node response can be dealt with one after another. Thus, the transmission of an “RTS” frame and reception of a “CTS” frame are two actions that have the same maximum single-trip propagation delay, Pmax. If we define the fixed length gap between a control frame and its consequent frame as “CML”, thus, the gap at the source between RTS and CTS is called CMLRTS, and the gap at the destination between “CTS” and “Data” is called “CMLCTS”. We define:

CMLRTS = CMLCTS = Pmax(1)

for the worst propagation scenario. After receiving the RTS frame, the destination then uses the distance information measured from the “RTS” frame to calculate the time to reply with a “CTS” frame so that the “CTS” frame reaches the source after a “CML” space can be counted. During the gap of “CML”, a potential interferer is avoided for collision-free transmission. Once the “Adjusted Response” state finishes, the source sends the data packets through the corresponding channel and goes to the “ACK” state. In summary, the second stage allows the destination to negotiate with the source, which gives both the source and the destination more flexibility and therefore reduces the chance that the destination fails due to channel collision.

The third stage starts as soon as the “CTS” frame is actually received. During this stage, if the destination receives “Data” from the source, it goes to the “Response adjustment” state to verify that the data packet is coming from the source. Otherwise, a timeout occurs.

At the fourth stage, “ACK” for the corresponding data packets are sent through the selected channel once the “Response adjustment” state finishes. After receiving the first ACK packet, the source finishes its transmission process. The BTS values are reset, and the node goes to a “Channel request” state if there are packets to transmit.

### 2.3. RBF Learning Network for Localization Errors Estimation

The sound propagation loss is one of the major reasons for cooperative localization errors. It is composed mainly of three aspects: namely, geometrical spreading, attenuation by absorption, and the anomaly of propagation:

(2) 10logA(l,f)=k⋅10logl+l⋅α

where α is the absorption coefficient in dB/km, k represents the geometrical spreading factor, l represents the transmission range, and f represents the signal frequency.

If we set $N_{t}$ as the turbulence noise, $N_{v}$ as the vehicle noise, $N_{w}$ as the wind driven wave noise, and $N_{th}$ as the thermal noise,

(3) $${N=N}_{t}+N_{v}+N_{w}{+N}_{th}$$

therefore, we obtain the channel capacity as:

(4) $$E_{trans}=\int_{B}{log}_{2}\left(1+\frac{P_{tx}}{A(l,f)NB}\right)df$$

where B is the bandwidth and $P_{tx}$ is the signal transmission power.

MAUVs in the formation should not only keep the formation configuration to realize purposed missions, but also avoid collision with obstacles. The formation shape and relative distances maintenance are important. If we set $p_{c}$ as the formation center, one obtains:

(5) $$p_{c}=\frac{1}{N}\sum_{i=1}^{N}p_{i}.$$

Each AUV can acquire a geometric center by communicating with its neighbors so as to keep the formation. Hence, the error between $p_{c}$ and the desired center, $p_{c}^{d}=$ ${\left[x_{c}^{d},y_{c}^{d},z_{c}^{d},\theta_{c}^{d},\psi_{c}^{d},\phi_{c}^{d}\right]}^{T}$ is the desired center of the formation region:

(6) $$e=p_{c}-p_{c}^{d}+{\hat{W}}_{i}^{T}\sigma (s_{i})$$

where ${\hat{W}}_{1,i}^{T}\sigma_{1,i}(s_{i})={\left[{\hat{W}}_{1,i}^{T}\sigma_{1,i}(s_{i}),{\hat{W}}_{2,i}^{T}\sigma_{2,i}(s_{i}),{\hat{W}}_{3,i}^{T}\sigma_{3,i}(s_{i})\right]}^{T}$ is the RBF neural network to estimate three dimensional cooperative localization errors caused by the data transmission packets loss and measurement noise. $\hat{W}=\left[w_{1},\ldots ,w_{N_{h}}\right]$ is the weight vector, while $s_{i}$ represents the input, including the packet loss, delay, current relative distance and between the AUVS, throughput, and current AUV speed. 

The output of the RBF neural network can be expressed in the following:

(7) $$f_{i}=\sum_{m=1}^{N_{h}}\left[w_{im}\bar{\sigma }(\sum_{k=1}^{N_{i}}\xi_{mk}\mu_{k}+\delta_{\xi j})+\delta_{wj}\right],\begin{array}{cc} & i=1,2,\ldots ,N_{o} \end{array}$$

where $N_{h}$, $N_{i}$, and $N_{o}$ represent the number of hidden layers, input layers, and output neurons. $w_{im}$ and $\xi_{mk}$ denote the network weights, $\delta_{\xi j}$ and $\delta_{wj}$ represent the threshold offsets, and $\bar{\sigma }()$ denotes the Gaussian function:

(8) $$\bar{\sigma }(\left\|s-r_{i}\right\|)=\exp(\frac{-{\left(s-r_{i}\right)}^{T}(s-r_{i})}{\gamma_{i}^{2}})$$

where $r_{i}$ is the center vector of the receptive field. $w_{im}$ can be obtained through the following reinforcement learning algorithm.

(9) $$w(s(t),a_{k}(t))=w(s(t),a_{k}(t))+\alpha \left[r(t+1)+\gamma w^{*}(s(t+1))-w(s(t),a_{k}(t))\right]$$

In this algorithm, the action is taken on the packets transmission episode. The actions are chosen through the ε greedy strategy. If ε>>0, the actions are taken randomly of $a(t)\in U(a_{\min},a_{\max})$. When ε<<1, the system exploits the knowledge through selecting the actions. The actions are selected through the comparisons between a random value of $x_{\varepsilon }\in U[0,1]$ and ε:

(10) $$a(t)=\{\begin{array}{c} U(a_{\min},a_{\max})\begin{array}{cc} & \begin{array}{cc} if & x_{\varepsilon }\leq \varepsilon \end{array} \end{array}\\ \arg\max_{a}w(s(t),a_{k}(t))\begin{array}{cc} & \begin{array}{cc} if & x_{\varepsilon }>\varepsilon \end{array} \end{array} \end{array}.$$

The actions represent the power transmission levels. The state is a combination of transmission energy $E_{trans}$ and channel transmission error evaluation, $P_{\mathrm{error}}$:

(11) $$\{\begin{array}{c} s(t)=\left\|E_{trans}\right\|+\left\|P_{\mathrm{error}}\right\|\\ P_{\mathrm{error}}=1-{\left(1-B_{error}\right)}^{N_{bit}} \end{array}$$

where $B_{error}$ is the bit error rate and $N_{bit}$ is the number of bits in the packet [13]. If each transmission action attempts to transmit the total packets, the rewards are defined as a combination of packets reception and energy power levels:

(12) $$r(t)=\pi [(pr^{q}(t)-1)n_{pts}+(n_{pts}-p_{Ediss}(t))-\frac{m_{pr}n_{pts}}{2}]$$

where π is the quantization step size factor between two consecutive quantization levels. pr(t) and $p_{Ediss}(t)$ are the packets reception levels and energy dissipation levels, respectively, while $m_{pr}$ is the number of quantized pr(t) levels.

If one defines

(13) $${\dot{p}}_{c}^{d}=L\left(G\right)e-p_{c}^{d}+p+1⊗\beta$$

where β is the maximum speed of desired trajectory $p^{d}$, $\beta =\max(p^{d})$, ⊗ is the Kronecker product.

Then, the derivative of the error is given by:

(14) $$\dot{e}=-\left(L\left(G\right)+I\right)e+\frac{1}{N}\sum_{j=1}^{N}{\dot{p}}_{j}+\frac{1}{N}\sum_{j=1}^{N}\left(p_{j}-p_{i}\right)-\beta +{\dot{\hat{W}}}_{1,i}^{T}\sigma_{1,i}(s_{i})$$

where ${\dot{\hat{W}}}_{1,i}^{T}=-\Gamma_{1,i}(\sigma (s_{i})\eta_{1,i}+\tau_{1,i}{\hat{W}}_{1,i}^{T})$. $\Gamma_{1,i}$, $\tau_{1,i}$, and $\eta_{1,i}$ are free parameters, $\eta_{1,i}={\left[\eta_{11,i},\eta_{12,i},\eta_{13,i}\right]}^{T}$.

### 2.4. Formation Shape Maintenance with Potential Field

**Figure 5:**
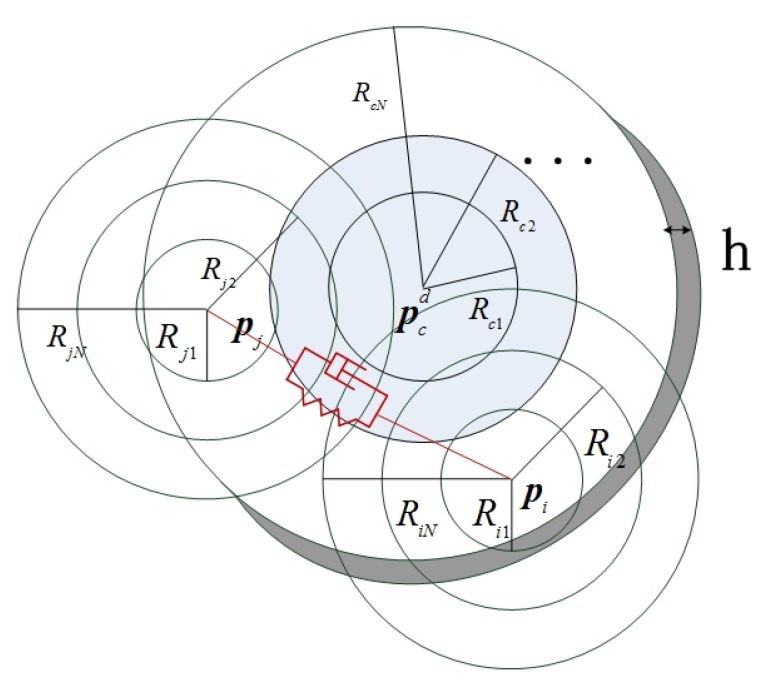
The layered region for AUV formation and collision avoidance.

Potential functions play a great role in helping AUVs move along the desired gradients directions and finally stabilize at the local minima. The following will define the layered potential functions’ shape for the AUVs to reach the desired region and maintain a formation shape (see Figure 5).

(15) $$f_{S}\left(\delta \eta_{i}\right)={\left[f_{S1}\left(\delta \eta_{io1}\right),f_{S2}\left(\delta \eta_{io2}\right),\ldots ,f_{Sm}\left(\delta \eta_{iom}\right)\right]}^{T}\leq 0$$

where $\eta_{iol}=\eta_{i}-\eta_{ol}$, $\eta_{ol}$ is a constant reference point of the l-th desired region, l=1,2,…,m, and m is the total number of objective functions. $f_{Sl}\left(\delta \eta_{iol}\right)$ represents the scalar functions with continuous partial derivatives. From Equation (1), the desired range of AUV motions in the formation is defined as a cylindrical and ring-shape region. For each AUV $p_{i}$, the desired region is the ring centered around $p_{c}^{d}$ between $R_{1}$ and $R_{2}$ with height h. Therefore, the scalar attractive forces of the shape function can be defined as follows.

(16) $$\begin{array}{l} \mathrm{Layer}\;1:\{\begin{array}{c} f_{S1}\left(\delta \eta_{io1}\right)={\left(x_{i}-x_{c}^{d}\right)}^{2}-{\left(y_{i}-y_{c}^{d}\right)}^{2}-R_{1}{}^{2}\leq 0\\ f_{S1}\left(\delta \eta_{io1}\right)={\left(z_{i}-z_{c}^{d}\right)}^{2}-h^{2}\leq 0\\ f_{S1}\left(\delta \eta_{io1}\right)={\left(\theta_{i}-\theta_{c}^{d}\right)}^{2}-\theta_{h}^{2}\leq 0 \end{array}\\ \mathrm{Layer}\;2:\{\begin{array}{c} \begin{array}{l} f_{S2}\left(\delta \eta_{io2}\right)=R_{1}{}^{2}-{\left(x_{i}-x_{c}^{d}\right)}^{2}-{\left(y_{i}-y_{c}^{d}\right)}^{2}\leq 0\\ and\begin{array}{cc} & f_{S2}\left(\delta \eta_{io2}\right)={\left(x_{i}-x_{c}^{d}\right)}^{2}-{\left(y_{i}-y_{c}^{d}\right)}^{2}-R_{2}{}^{2}\leq 0 \end{array} \end{array}\\ f_{S2}\left(\delta \eta_{io2}\right)={\left(z_{i}-z_{c}^{d}\right)}^{2}-h^{2}\leq 0\\ f_{S2}\left(\delta \eta_{io2}\right)={\left(\theta_{i}-\theta_{c}^{d}\right)}^{2}-\theta_{h}^{2}\leq 0 \end{array}\\ \mathrm{Layer}\;3:\{\begin{array}{c} \begin{array}{l} f_{S3}\left(\delta \eta_{io2}\right)=R_{2}{}^{2}-{\left(x_{i}-x_{c}^{d}\right)}^{2}-{\left(y_{i}-y_{c}^{d}\right)}^{2}\leq 0\\ and\begin{array}{cc} & f_{S3}\left(\delta \eta_{io2}\right)={\left(x_{i}-x_{c}^{d}\right)}^{2}-{\left(y_{i}-y_{c}^{d}\right)}^{2}-R_{3}{}^{2}\leq 0 \end{array} \end{array}\\ f_{S3}\left(\delta \eta_{io2}\right)={\left(z_{i}-z_{c}^{d}\right)}^{2}-h^{2}\leq 0\\ f_{S3}\left(\delta \eta_{io2}\right)={\left(\theta_{i}-\theta_{c}^{d}\right)}^{2}-\theta_{h}^{2}\leq 0 \end{array} \end{array}$$

Hence, the center of the desired formation region is:

(17) $$p_{c}^{d}={\left[r_{c}^{d},z_{c}^{d},\theta_{c}^{d}\right]}^{T}.$$

If $k_{l}$ is set as a positive constant, the traditional potential energy function for the desired formation regions in Figure 5 is:

(18) $$P_{Sl}\left(\delta \eta_{iol}\right)=\frac{k_{l}}{2}{\left[\max(0,f_{Sl}\left(\delta \eta_{iol}\right))\right]}^{2}=\{\begin{array}{ll} 0 & f_{Sl}\left(\delta \eta_{iol}\right)\leq 0\\ \frac{k_{l}}{2}f_{Sl}^{2}\left(\delta \eta_{iol}\right) & f_{Sl}\left(\delta \eta_{iol}\right)>0 \end{array}.$$

In the consideration with the under-actuated characteristic of AUV, the potential energy functions’ magnitude produced from three-dimensional distances have been reduced to improve the scheme robustness and convergence. On the other hand, since the rudder angle is significant for under-actuated AUV to arrive at desired positions, the yaw error of AUV formation appears to be more important.

(19) $$\{\begin{array}{c} P_{Sm}\left(\delta p_{iom}\right)=\frac{k_{m}}{2}\left[\max(0,In(\left|f_{Sm}\left(\delta p_{iom}\right)\right|)\right]=\{\begin{array}{ccc} 0 & f_{Sm}\left(\delta p_{iom}\right)\leq 0 & \\ \frac{k_{m}}{2}In(\left|f_{Sm}\left(\delta p_{iom}\right)\right|) & f_{Sm}\left(\delta p_{iom}\right)>0 & m=1,2,3\ldots \end{array}\\ P_{Sm}\left(\delta p_{iom}\right)=\frac{k_{m}}{2}{\left[\max(0,f_{Sm}\left(\delta p_{iom}\right))\right]}^{2}=\{\begin{array}{ccc} 0 & f_{Sm}\left(\delta p_{iom}\right)\leq 0 & \\ \frac{k_{m}}{2}f_{Sm}^{2}\left(\delta p_{iom}\right) & f_{Sk}\left(\delta p_{iom}\right)>0 & m=4 \end{array} \end{array}$$

Thus, the region error for the i-th AUV is defined as follows.

(20) $$\begin{array}{l} {\left(\frac{\partial P_{Si}\left(\delta p_{iok}\right)}{\partial p_{i}}\right)}^{T}\\ =\sum_{m=1}^{N}\left(k_{m}\frac{1}{\max\left(0,f_{Sm}\left(\delta p_{iom}\right)\right)}\times {\left(\frac{\partial f_{Sm}\left(\delta p_{iom}\right)}{\partial \delta q_{iom}}\right)}^{T}\right)+k_{4}\max\left(0,f_{S4}\left(\delta p_{io4}\right)\right)\times {\left(\frac{\partial f_{Sk}\left(\delta p_{io4}\right)}{\partial \delta p_{io4}}\right)}^{T}=\Delta \xi_{i} \end{array}$$

For the collision avoidance conditions, the repulsive forces between AUVs or AUVs and obstacles are defined in the form as:

(21) $$f_{irep}=k_{rep}\left((\frac{1}{\left\|p_{i}-p_{j}\right\|})\frac{p_{i}-p_{j}}{\left\|p_{i}-p_{j}\right\|}+(\frac{1}{\left\|p_{i}-p_{oi}\right\|})\frac{p_{i}-p_{oi}}{\left\|p_{i}-p_{oi}\right\|}\right)$$

where $p_{oi}$ is the position vector of the i-th obstacle, the energy functions are defined on the basis of the collision avoidance region:

(22) $$\{\begin{array}{c} g_{1ij}\left(\delta \eta_{ij}\right)=R_{i1}^{2}-{\left\|\delta \eta_{ij}\right\|}^{2}\leq 0\\ g_{2ij}\left(\delta \eta_{ij}\right)=R_{i2}^{2}-{\left\|\delta \eta_{ij}\right\|}^{2}\leq 0\\ \vdots \\ g_{Nij}\left(\delta \eta_{ij}\right)=R_{iN}^{2}-{\left\|\delta \eta_{ij}\right\|}^{2}\leq 0 \end{array}$$

where $\delta \eta_{ij}=\eta_{i}-\eta_{j}$, $g_{1ij},g_{2ij},\ldots ,g_{NLij}$ are the functions for the first layer, second layer,…, and the innermost layer, respectively, and these layers are continuous and differentiable, while N is the number of layers, and $R_{i1}>R_{i2}>\ldots R_{iN}$ denote the radius of the first, second, and innermost layers, respectively.

Similar to the equations shown in (19), the collision avoidance energy functions have been magnitude reduced as:

(23) $$\{\begin{array}{c} Q_{1ij}\left(\delta p_{ij}\right)=\frac{k_{1ij}}{2}\left[\max(0,In\left|g_{1ij}\left(\delta p_{ij}\right)\right|)\right]\\ Q_{2ij}\left(\delta p_{ij}\right)=\frac{k_{2ij}}{2}\left[\max(0,In\left|g_{2ij}\left(\delta p_{ij}\right)\right|)\right]\\ \vdots \\ Q_{Nij}\left(\delta p_{ij}\right)=\frac{k_{Nij}}{2}\left[\max(0,In\left|g_{Nij}\left(\delta p_{ij}\right)\right|)\right] \end{array}$$

where $k_{Nij}>\cdots >k_{2ij}>k_{1ij}$ are positive constants. The potential energy for collision avoidance between the i-th and j-th vehicle is:

(24) $$\begin{array}{l} Q_{ij}\left(\delta p_{ij}\right)=Q_{1ij}\left(\delta p_{ij}\right)+Q_{2ij}\left(\delta p_{ij}\right)+\ldots +Q_{Lij}\left(\delta p_{ij}\right)\\ =\frac{k_{1ij}}{2}{\left[\max(0,In\left|g_{1ij}\left(\delta p_{ij}\right)\right|)\right]}^{2}+\frac{k_{2ij}}{2}{\left[\max(0,In\left|g_{2ij}\left(\delta p_{ij}\right)\right|)\right]}^{2}+\ldots +\frac{k_{Nij}}{2}{\left[\max(0,In\left|g_{Nij}\left(\delta p_{ij}\right)\right|)\right]}^{2} \end{array}$$

and

(25) $$\frac{\partial Q_{Nij}\left(\delta q_{1ij}\right)}{\partial \delta q_{ij}}=\sum_{h=1}^{N}k_{hij}\max\left(0,\frac{1}{g_{hij}\left(\delta q_{ij}\right)}\right)\times {\left(\frac{\partial g_{hij}\left(\delta q_{ij}\right)}{\partial \delta q_{ij}}\right)}^{T}≜\Delta \rho_{ij}.$$

Therefore, if $p_{c}^{d}={\left[x_{c}^{d},y_{c}^{d},z_{c}^{d},\phi_{c}^{d},\psi_{c}^{d},\theta_{c}^{d}\right]}^{T}$ is set as the desired center of AUV formation, the desired AUV positions and formation shape can be obtained through Equations (16)–(25). 

## 3. Adaptive RBF Formation Scheme

The dynamic equation of the i-th AUV can be expressed as:

(26) $$M_{i}(p_{i}){\ddot{p}}_{i}+C_{i}(p_{i}){\dot{p}}_{i}+D_{i}(p_{i}){\dot{p}}_{i}+g_{i}(p_{i})+\Delta_{i}(p_{i})=T_{i}$$

where $M_{i}(p_{i})$ is the 6×6 mass matrix of the AUV, $C_{i}(p_{i})$ is a 6×6 matrix of centrifugal and coriolis terms, $D_{i}(p_{i})$ is the damping matrix, $g_{i}(p_{i})$ is the vector of gravitational forces and moments, $\Delta_{i}(p_{i})$ is uncertain dynamics, and $T_{i}$ contains the forces and torques from thrusts. If we define:

(27) $${\dot{p}}_{ci}={\dot{p}}_{c}^{d}-(\alpha_{i}\Delta \xi_{i}+\gamma \sum_{j=1}^{N_{i}}\Delta \rho_{ij})$$

and set $\Delta \varepsilon_{i}=\alpha_{i}\Delta \xi_{i}+\gamma \sum_{j=1}^{N_{i}}\Delta \rho_{ij}$, we have ${\dot{p}}_{ci}={\dot{p}}_{c}^{d}-\Delta \varepsilon_{i}$, where ${\ddot{p}}_{ci}={\dot{p}}_{c}^{d}-\Delta {\dot{\varepsilon }}_{i}$. We define a sliding vector for the i-th AUV as:

(28) $$s_{i}={\dot{p}}_{i}-{\dot{p}}_{ci}={\dot{p}}_{i}-{\dot{p}}_{c}^{d}+\Delta \varepsilon_{i}.$$

Thus, we obtain:

(29) $${\dot{s}}_{i}={\ddot{p}}_{i}-{\ddot{p}}_{c}^{d}+\Delta {\dot{\varepsilon }}_{i}.$$

Substituting Equations (28) and (29) into Equation (27), one has:

(30) $$M_{i}(p_{i}){\dot{s}}_{i}+C_{i}(p_{i})s_{i}+D_{i}(p_{i})s_{i}+M_{i}(p_{i}){\ddot{p}}_{c}^{d}+C_{i}(p_{i}){\dot{p}}_{c}^{d}+D_{i}(p_{i}){\dot{p}}_{c}^{d}+g_{i}(p_{i})+\Delta_{i}(p_{i})=T_{i}.$$

According to the adaptive control principle, we obtain:

(31) $$M_{i}(p_{i}){\ddot{p}}_{c}^{d}+C_{i}(p_{i}){\dot{p}}_{c}^{d}+D_{i}(p_{i}){\dot{p}}_{c}^{d}+g_{i}(p_{i})+\Delta_{i}(p_{i})={ϒ}_{i}\left(p_{i},{\dot{p}}_{i},{\dot{p}}_{c}^{d},{\ddot{p}}_{c}^{d}\right)\lambda_{i}$$

where ${ϒ}_{i}\left(p_{i},{\dot{p}}_{i},{\dot{p}}_{c}^{d},{\ddot{p}}_{c}^{d}\right)$ is a known regressor matrix and $\lambda_{i}$ represents the dynamic parameters. Therefore, the RBF-based region based adaptive controller is:

(32) $$T_{i}=-K_{si}s_{i}-K_{p}\Delta \varepsilon_{i}+{ϒ}_{i}\left(p_{i},{\dot{p}}_{i},{\dot{p}}_{c}^{d},{\ddot{p}}_{c}^{d}\right){\hat{\lambda }}_{i}+{\hat{W}}_{i}^{T}\sigma (s_{i}).$$

If we set $L_{i}$ as positive definite matrices, the estimated parameter ${\hat{\lambda }}_{i}$ is updated as:

(33) $${\hat{\lambda }}_{i}=-L_{i}{ϒ}_{i}^{T}\left(q_{i},{\dot{q}}_{i},{\dot{q}}_{c}^{d},{\ddot{q}}_{c}^{d}\right)s_{i}.$$

Therefore,

(34) $$M_{i}(q_{i}){\dot{s}}_{i}+C_{i}(q_{i})s_{i}+D_{i}(q_{i})s_{i}+K_{si}s_{i}+K_{p}\Delta \varepsilon_{i}+{ϒ}_{i}\left(q_{i},{\dot{q}}_{i},{\dot{q}}_{c}^{d},{\ddot{q}}_{c}^{d}\right)\Delta \lambda_{i}+{\hat{W}}_{i}^{T}\sigma (s_{i})=0$$

where $\Delta \lambda_{i}=\lambda_{i}-{\hat{\lambda }}_{i}$. 

In order to prove the stability of the RBF-based adaptive formation scheme, we obtain a Lyapunov-like function for the multiple AUVs system as:

(35) $$\begin{array}{cc} V= & \sum_{i=1}^{N}\frac{1}{2}s_{i}^{T}M_{i}(q_{i})s_{i}+\sum_{i=1}^{N}\frac{1}{2}\Delta \lambda_{i}^{T}M_{i}(q_{i})\lambda_{i}+\sum_{k=1}^{3}\frac{1}{2}{\tilde{W}}_{k,i}^{T}\Gamma_{k,i}^{-1}{\tilde{W}}_{k,i}^{}\\ & +\sum_{i=1}^{N}\frac{1}{2}\alpha_{i}K_{p}\sum_{l=1}^{6}K_{l}P_{Sm}\left(\delta q_{lom}\right)+\sum_{i=1}^{N}\frac{1}{2}\gamma_{i}K_{p}\sum_{j=1}^{N}K_{ij}Q_{2ij}\left(\delta q_{ij}\right) \end{array}$$

We obtain from Equations (20), (31), and (32):

(36) $$\begin{array}{l} {\dot{V}}_{i}=-\sum_{i=1}^{N}s_{i}^{T}K_{si}s_{i}^{}-\sum_{i=1}^{N}s_{i}^{T}D_{i}(q_{i})s_{i}^{}-\sum_{i=1}^{N}s_{i}^{T}K_{p}\Delta \varepsilon_{i}+\sum_{i=1}^{N}\alpha_{i}K_{p}{\dot{e}}_{}^{T}\Delta \xi_{i}\\ +\sum_{i=1}^{N}\frac{1}{2}\gamma_{i}K_{p}\sum_{j=1}^{N_{i}}\sum_{h=1}^{L}k_{hij}\delta {\dot{q}}_{ij}^{T}\left[\max(0,g_{hij}\left(\delta q_{ij}\right))\right]{\left(\frac{\partial g_{hij}\left(\delta q_{ij}\right)}{\partial \delta q_{ij}}\right)}^{T}-\sum_{k=1}^{3}\frac{1}{2}{\tilde{W}}_{k,i}^{T}(\sigma (s_{i})\eta_{k,i}+\tau_{k,i}{\hat{W}}_{k,i}^{T}) \end{array}$$

If we set $E_{N_{i}}={\left[\underset{N_{i}}{\underset{︸}{1,\ldots ,1}}\right]}^{T}$, the last term of the Equation (36) can be rewritten by using Equation (25):

(37) $$\sum_{i=1}^{N}\frac{1}{2}\gamma_{i}\sum_{j=1}^{N_{i}}K_{p}\dot{e}\Delta \rho_{ij}-\sum_{i=1}^{N}\frac{1}{2}\gamma_{i}K_{p}\sum_{j=1}^{N_{i}}\sum_{h=1}^{L}k_{hij}{\dot{e}}^{T}\left[\max(0,g_{hij}\left(\delta q_{ij}\right))\right]{\left(\frac{\partial g_{hij}\left(\delta q_{ij}\right)}{\partial \delta q_{ij}}\right)}^{T}.$$

From Equation (22), we can obtain

(38) $$g_{hij}\left(\delta q_{ij}\right)=g_{hji}\left(\delta q_{ji}\right)\begin{array}{ccc} & and & \end{array}\frac{\partial g_{hij}\left(\delta q_{ij}\right)}{\partial \delta q_{ij}}=-\frac{\partial g_{hji}\left(\delta q_{ji}\right)}{\partial \delta q_{ji}}.$$

Thus, the last term of Equation (35) can be written as

(39) $$\begin{array}{l} \sum_{i=1}^{N}\frac{1}{2}\gamma_{i}K_{p}\sum_{j=1}^{N_{i}}\sum_{h=1}^{L}k_{hij}{\dot{e}}^{T}\left[\max(0,g_{hji}\left(\delta q_{ji}\right))\right]{\left(\frac{\partial g_{hji}\left(\delta q_{ji}\right)}{\partial \delta q_{ji}}\right)}^{T}\\ =\sum_{i=1}^{N}\frac{1}{2}\gamma_{i}K_{p}\sum_{j=1}^{N_{i}}\sum_{h=1}^{L}k_{hji}{\dot{e}}^{T}\left[\max(0,g_{hji}\left(\delta q_{ji}\right))\right]{\left(\frac{\partial g_{hji}\left(\delta q_{ji}\right)}{\partial \delta q_{ji}}\right)}^{T}\\ =\sum_{i=1}^{N}\frac{1}{2}\gamma_{i}K_{p}\sum_{j=1}^{N_{i}}\sum_{h=1}^{L}k_{hji}{\dot{e}}^{T}\left[\max(0,g_{hji}\left(\delta q_{ji}\right))\right]{\left(\frac{\partial g_{hji}\left(\delta q_{ji}\right)}{\partial \delta q_{ji}}\right)}^{T}\\ =\sum_{i=1}^{N}\frac{1}{2}\gamma_{i}K_{p}\sum_{j=1}^{N_{i}}{\dot{e}}^{T}\Delta \rho_{ji}\\ =\sum_{i=1}^{N}\frac{1}{2}\gamma_{i}K_{p}\sum_{j=1}^{N_{i}}{\dot{e}}^{T}\Delta \rho_{ij} \end{array}$$

Moreover, $-\tau_{k,i}{\tilde{W}}_{k,i}^{}{\hat{W}}_{k,i}^{T}\leq -\frac{1}{2}\tau_{k,i}({\left\|{\tilde{W}}_{k,i}^{}\right\|}^{2}+{\left\|{\tilde{W}}_{k,i}^{*}\right\|}^{2})$, $W_{k,i}^{*}$ denotes the ideal constant weights. 

Therefore, the time derivative of the Lyapunov function in Equation (37) is

(40) $${\dot{V}}_{i}\leq -\sum_{i=1}^{N}s_{i}^{T}K_{si}s_{i}^{}-\sum_{i=1}^{N}s_{i}^{T}D_{i}(q_{i})s_{i}^{}-\sum_{i=1}^{N}K_{p}\Delta \varepsilon_{i}^{T}\Delta \varepsilon_{i}-\frac{1}{2}\tau_{k,i}({\left\|{\tilde{W}}_{k,i}^{}\right\|}^{2}+{\left\|{\tilde{W}}_{k,i}^{*}\right\|}^{2})\leq 0.$$

From Equation (40), it can be obtained that $s_{i}$, $\Delta \varepsilon_{i}$, $\Delta {\dot{\xi }}_{i}$, $\Delta {\dot{\rho }}_{ij}$ and $\Delta {\dot{\varepsilon }}_{i}$ are bounded. ${\ddot{q}}_{ri}$ is bounded if $\ddot{e}$ is bounded. Thus, ${\dot{s}}_{i}$ is bounded from Equation (32). Applying Barbalat’s lemma, we obtain and $s_{i}\to 0$ as t→∞ if $\dot{e}\to 0$. From Equation (28), $\Delta \rho_{ij}\to 0$.

Since

(41) $$\Delta \varepsilon_{i}=\alpha_{i}\Delta \xi_{i}+\gamma \sum_{j=1}^{N_{i}}\Delta \rho_{ij}\to 0$$

as t→∞, all the error terms are summing yields:

(42) $$\sum_{i=1}^{N}(\alpha_{i}\Delta \xi_{i}+\gamma \sum_{j=1}^{N_{i}}\Delta \rho_{ij})\to 0$$

Since the interactive forces between AUVs are bi-directional, the summation of all the interactive forces in the systems is zero, we obtain:

(43) $$\sum_{i=1}^{N}\alpha_{i}\Delta \xi_{i}\to 0.$$

One trivial solution of Equation (43) is $\Delta \xi_{i}\to 0$, which means that all the AUVs remain in the desired region all the time because of Equation (40). This means that each AUV is in the desired region and maintains a minimum distance among themselves simultaneously. On the contrary, if we assume $\Delta \xi_{i}\neq 0$, the AUV are outside the desired region. Thus, some of the AUVs must be on the opposite sides of the desired region and their $\Delta \xi_{i}$ values can not be cancelled out, which contradicts with the fact that $\sum_{i=1}^{N}\alpha_{i}\Delta \xi_{i}=0$. Therefore, the only possibility is $\sum_{i=1}^{N}\alpha_{i}\Delta \xi_{i}=0$ when $\Delta \xi_{i}=0$. From Equation (41), $\Delta \rho_{ij}=0$. Therefore, if and only if all the forces of $\Delta \xi_{i}$ are zero or cancelled out, does $\sum_{i=1}^{N}\alpha_{i}\Delta \xi_{i}=0$. This means that some AUVs must be on the opposite sides of the desired region. When there are interactions or coupling among the AUVs from different sides of the desired region, a reasonable weightage can be obtained for $\Delta \xi_{i}$ by adjusting $\alpha_{i}$. Finally, since $s_{i}\to 0$ and $\Delta \xi_{i}\to 0$, we can conclude from Equation (28) that $\Delta \rho_{ij}\to 0$. Hence, all the AUVs are synchronized to the same speed and maintain constant distances among themselves at steady state.

## 4. Simulations and Experiments

**Figure 6:**
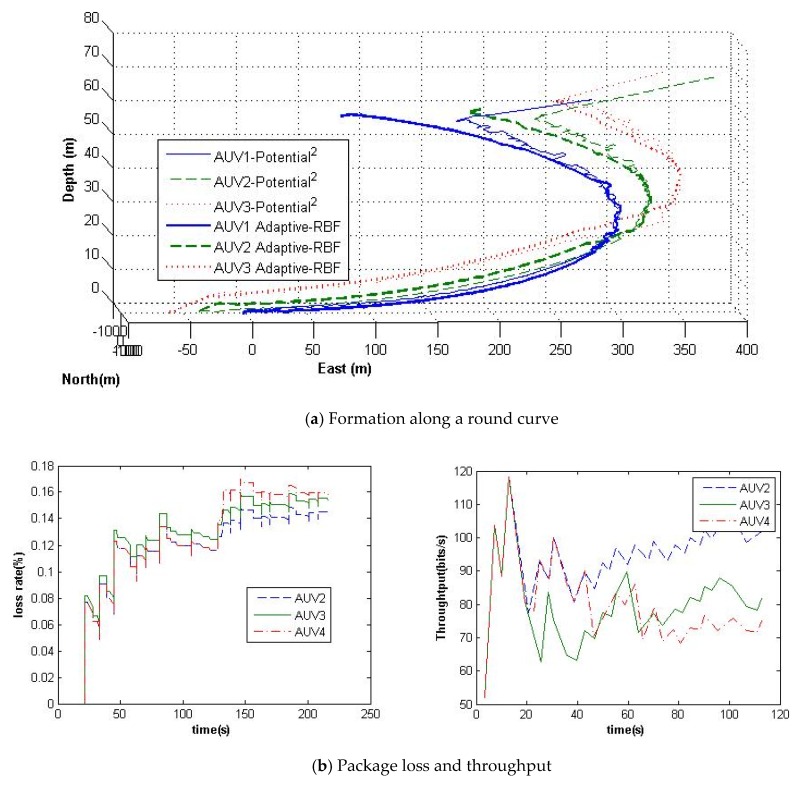

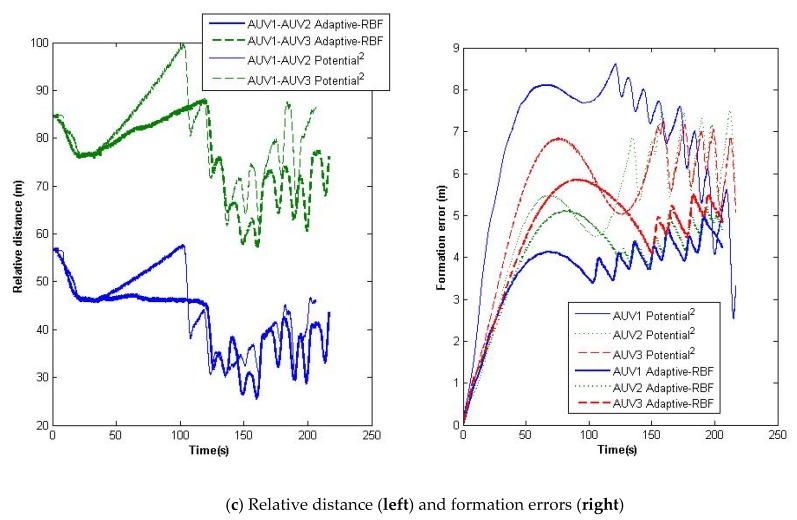
Formation simulation along a round curve.

**Figure 7:**
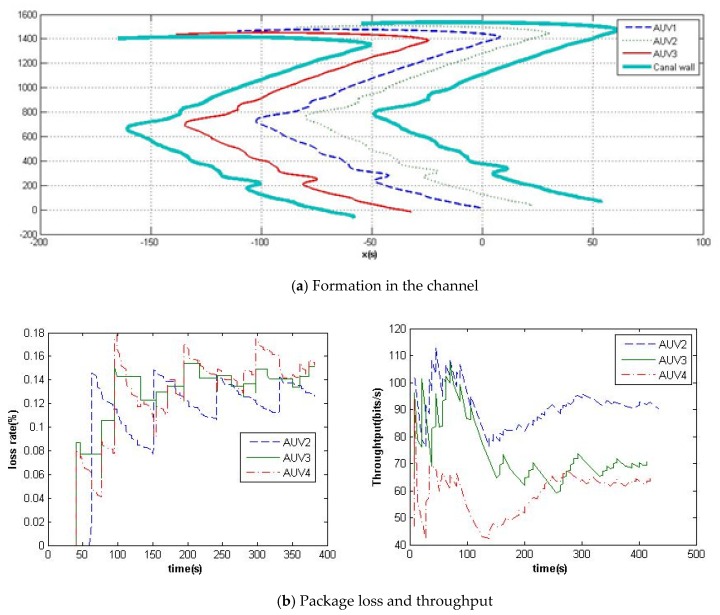
Formation simulation in the channel.

In order to analyze and verify the designed communication link framework and formation scheme, simulations and experiments have been launched. In the formation simulations of Figure 6 and Figure 7, comparisons have been made on the proposed adaptive formation scheme with and without the RBF neural network. The disturbance is set with a current speed as 0.1 m/s in the west direction. The simulation includes the formation along a round curve and cruising in the confined channel. Their communications are simulated in the NS-2 simulator on the basis of the communication protocol of Section 2. The formation control simulation platform was established on the basis of AUV hydrodynamic equations. 

In Figure 6, the three AUVs are planned to follow a round curve with a line shape, e.g., the followers are planned to maintain the same distance one after another. The protocol for linear topology has been applied for the formation communication on the basis of the network framework of Section 2. Since the radius of the trace curvature is greater than the radius of the AUVs’ gyration, these three AUVs can keep formation cruising precisely. The package loss and data transmission throughput are illustrated in Figure 6b; one can improve the cooperative localization accuracy through reinforcement learning RBF neural network and therefore improve the formation stability. From Figure 6c, the reinforcement learning RBF neural network can compensate and reduce the cooperative localization errors caused by communication loss through Equations (12)–(14).

Channel cooperative exploration is one of the important applications, and it is very difficult for MAUVs because of the change of channel size and curve. Through the reinforcement learning RBF neural network, the MAUVs’ formation can obtain more accurate cooperative localization information. The multibody system-based potential field can help MAUVs maintain and change their formation shape according to the environment. The protocol for one–many contending topology and linear topology have been applied and switched according to the shape requirements. 

**Figure 8:**
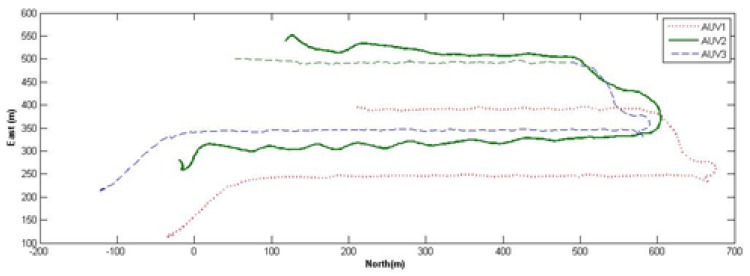
Formation coverage experiments.

Offshore experiments of MAUVs formation coverage exploration are illustrated in Figure 8. The vehicles were given folding lines with a 90-degree yaw path to test the formation performance of heterogeneous AUVs. The three AUVs can keep their formation while cruising under the strategies proposed in this study.

## 5. Conclusions

MAUVs’ formation is of great significance for marine surveys and exploration. In order to realize MAUVs’ formation, this study has focused on their communication and formation. On the basis of the multibody system concept, the MAUVs’ formation and communication link framework has been established with an adaptive RBF strategy. The connection for communication and formation between AUVs can be viewed as a springs and damping system. The packets transmission scheme has been designed with multi-layered network topology, which reduces the packets’ loss rate and improves the throughput of the network. Moreover, through the reinforcement-learning RBF neural networks, an adaptive RBF formation strategy can be improved with more accurate cooperative localization information. Simulations and offshore experiments with multiple heterogeneous under-actuated AUVs testify the performance of proposed method.
